# Deep Convolutional Neural Network With a Multi-Scale Attention Feature Fusion Module for Segmentation of Multimodal Brain Tumor

**DOI:** 10.3389/fnins.2021.782968

**Published:** 2021-11-26

**Authors:** Xueqin He, Wenjie Xu, Jane Yang, Jianyao Mao, Sifang Chen, Zhanxiang Wang

**Affiliations:** ^1^School of Informatics, Xiamen University, Xiamen, China; ^2^Department of Cognitive Science, University of California, San Diego, San Diego, CA, United States; ^3^Department of Neurosurgery, The First Affiliated Hospital of Xiamen University, Xiamen, China; ^4^Xiamen Key Laboratory of Brain Center, Department of Neurosurgery, The First Affiliated Hospital of Xiamen University, Xiamen, China; ^5^Department of Neuroscience, School of Medicine, Institute of Neurosurgery, Xiamen University, Xiamen, China

**Keywords:** magnetic resonance imaging (MRI), semantic segmentation, convolutional neural network, residual network, attention mechanism, brain tumor

## Abstract

As a non-invasive, low-cost medical imaging technology, magnetic resonance imaging (MRI) has become an important tool for brain tumor diagnosis. Many scholars have carried out some related researches on MRI brain tumor segmentation based on deep convolutional neural networks, and have achieved good performance. However, due to the large spatial and structural variability of brain tumors and low image contrast, the segmentation of MRI brain tumors is challenging. Deep convolutional neural networks often lead to the loss of low-level details as the network structure deepens, and they cannot effectively utilize the multi-scale feature information. Therefore, a deep convolutional neural network with a multi-scale attention feature fusion module (MAFF-ResUNet) is proposed to address them. The MAFF-ResUNet consists of a U-Net with residual connections and a MAFF module. The combination of residual connections and skip connections fully retain low-level detailed information and improve the global feature extraction capability of the encoding block. Besides, the MAFF module selectively extracts useful information from the multi-scale hybrid feature map based on the attention mechanism to optimize the features of each layer and makes full use of the complementary feature information of different scales. The experimental results on the BraTs 2019 MRI dataset show that the MAFF-ResUNet can learn the edge structure of brain tumors better and achieve high accuracy.

## Introduction

In daily life, the human brain is the controller of all behaviors and the sender of activity instructions. As the main part of the human brain, the cerebrum is the highest part of the central nervous system. Brain health has an important impact on the human body. The brain tumor is one of the most common brain diseases and can be induced at any age. Therefore, the prevention of brain tumors is a significant part of daily health management. Brain tumors can be divided into glioma, meningioma, pituitary adenoma, schwannoma congenital tumor, and so on, among which glioma accounts for the largest proportion. More than half of gliomas are malignant tumors, among which glioblastoma is the most common malignant tumor of the brain and central nervous system, accounting for about 14.5% of all tumors (Ostrom et al., 2020). According to the World Health Organization (WHO) criteria, gliomas are classified into four grades, and the higher the grade, the more likely the tumor is to be malignant. Among them, grade I and II gliomas are low-grade gliomas (LGG), while grade III and IV gliomas are high-grade gliomas (HGG; Louis et al., 2007), which are malignant tumors. Brain tumors can cause serious damage not only to the brain but also to other parts of the body, such as vision loss, motor problems, sensory problems, and even shock in severe cases. Therefore, early detection of brain tumors and early intervention are the only way to minimize the impact of brain tumors.

In clinical medicine, brain tumor screening and diagnosis mainly involve physical examination, imaging examination, and pathological examination, among which physical examination is a preliminary diagnosis of the patient’s condition through a comprehensive physical examination by the doctor. The results are somewhat accidental, and it is impossible to accurately judge the condition. However, the pathological examination requires anesthesia operation to collect samples from patients, which is complicated, costly, and has certain damage to the patient’s body. Compared with the previous two methods, medical imaging has the characteristics of objectivity, accuracy, convenience, and low cost. It not only overcomes the inaccuracy and subjectivity of physical examination but also omits the cumbersome collection of biopsy samples in the pathological examination. And it is one of the main methods of auxiliary diagnosis for patients with brain tumors. The medical imaging techniques used to diagnose brain tumors mainly include magnetic resonance imaging (MRI) and computer tomography (CT). MRI images are clearer than CT. Especially for small tumors, the use of CT technology is prone to miss the diagnosis. And for soft tissues, the resolution of CT is much lower than that of MRI. Thus, the results of auxiliary diagnosis and treatment using MRI will be more accurate. In addition, CT imaging requires prior injection of radioactive isotopes into the patient, which can affect the human body to a certain extent. As a non-invasive and low-cost medical imaging technology, MRI has become the first choice for brain tumors diagnosis. In this article, MRI images are utilized as a data carrier to study the segmentation of glioma, which has the greatest risk of malignancies in brain tumors.

The number of brain tumor patients is increasing with the development of society, the accelerated pace of life, and the increase of people’s work pressure. Faster and more accurate intervention is the key to reduce the mortality rate of brain tumor-related diseases. During the analysis of the brain images, accurate identification of tumor area is the premise of subsequent qualitative diagnosis. However, large spatial and structural variability and low image contrast are the main problems in brain tumor segmentation.

Traditional brain imaging diagnosis mainly relies on manual analysis by professional doctors, which requires a lot of time and cost. With the huge and increasing amount of medical image data, the speed of manual analysis is far behind the speed of data generation. At the same time, due to the professional knowledge requirements of manual segmentation of brain tumors, the differences and workload of manual segmentation results, machine-participated semi-automatic or fully automatic brain tumor segmentation shows obvious advantages (Gordillo et al., 2013). In early studies, it was mainly aimed at semi-automatic segmentation of brain tumors (Gordillo et al., 2013). The purpose of semi-automatic segmentation research is to minimize human intervention when machines and humans work together to achieve the desired segmentation effect. But it is still affected by differences in human subjective consciousness. The automatic method exploits the model and prior knowledge to achieve independent segmentation.

Segmentation methods for brain tumors can be divided into four categories, which are threshold-based, region-based, classification-based, and model-based methods (Gordillo et al., 2013). Dawngliana et al. (2015) combined initial segmentation of multi-layer threshold with the morphological operation of level set to extract fine images. Since brain tumors are relatively easy to identify compared with other brain tissues, the characteristics of tumor regions can be extracted during the preprocessing stage, so that brain tumors can be segmented using region-based methods. Harati et al. (2011) proposed an improved scale-based fuzzy connectedness algorithm that automatically selects seed points on the scale. The method performed well in low-contrast tumor areas. Region-based methods are greatly affected by image pixel coherence, and noise or intensity changes may lead to holes or excessive segmentation (Gordillo et al., 2013). Another relatively similar idea is based on the prominent characteristics of brain tumors in medical images, which is to segment brain tumors based on tumor contour by feature extraction of brain tumor boundary information (Bauer et al., 2013). Essadike et al. (2018) determined the initial contour by using a tumor filter in analog optics and utilized this initial contour to define the active contour model to determine the tumor boundary. Ma et al. (2018) combined random forest and active contour models to automatically infer glioma structure from multimodal volumetric MR images and proposed a new multiscale patch-driven active contour model to refine the results using sparse representation techniques. In addition, due to the different formation mechanisms and surface features of different brain tumors, many researchers have studied the texture features of different brain tumors, and achieve tumor segmentation through voxel classification or clustering. Among the segmentation methods based on classification, Fuzzy C-means (FCM) is one of the mainstream methods because of its advantages in preserving the original image information. In the early stage, Pham et al. (1997) and Xu et al. (1997) applied the FCM method to MRI segmentation (Latif G. et al., 2021). Subsequently, many variants of standard FCM, such as bias-corrected FCM (BCFCM), enhanced FCM (EFCM), kernelized FCM (KFCM), and spatially constrained KFCM (SKFCM) emerged (Latif G. et al., 2021). However, the FCM method is easily disturbed by noise and has a high computational cost. Model-based methods include parametric deformable models, geometric deformable models or level sets, and so on. The above active contour models belong to the parameter deformable model. However, parametric deformable models are difficult to deal with topology changes of contour segmentation and merger naturally, so geometric deformable models or level sets are introduced (Gordillo et al., 2013). Lee et al. (2012) exploited the surface evolution principle of geometric deformation model and level set to achieve medical volume image segmentation and carried out tests on tumor tissues, but the computational efficiency of this method was low.

With the rise of deep learning, researchers began to apply deep networks to the automatic segmentation of brain tumors. Havaei et al. (2017) proposed a brain tumor segmentation model based on a deep neural network, which utilized local features and more global context features to learn the unique features of brain tumor segmentation. For images, a convolutional neural network shows obvious superiority. Pereira et al. (2016) designed a deeper network for glioma segmentation based on a convolutional neural network, using small kernels. Through intensity normalization and data enhancement, the segmentation effect of the network can be improved and the over-fitting can be avoided while the network parameters are minimized. Among them, U-Net (Ronneberger et al., 2015), as the classical model of the convolutional neural network, has outstanding application effect in medical images, so it is also widely used in MRI brain tumor segmentation, and there are many modifications based on U-Net. Latif U. et al. (2021) improved the automatic segmentation process of brain tumors by introducing size variability into the convolutional neural network and proposed a multi-inception-UNET model to improve the scalability of U-Net. Zhang et al. (2020) proposed a new type of densely connection inception convolutional neural network on the basis of U-Net architecture which was applied to medical images, and conducted experiments in tumor segmentation of brain MRI. They added the Inception-Res module and the densely connecting convolutional module to increase the width and depth of the network, and at the same time led to an increase in the number of parameters, which slows down the speed of model training data (Angulakshmi and Deepa, 2021).

In this article, a deep convolutional neural network composed of a U-Net and a multi-scale attention feature fusion module (MAFF) is proposed to achieve automatic segmentation of gliomas in 3D brain MRI images. By using multi-modal MRI data, high-precision segmentation of three tumor types is realized. The main contributions of this work are as follows:

(1) We introduce five residual connections to U-Net, which enhance the feature extraction ability of encoder blocks, the speed of network convergence, and alleviate the gradient vanishing problem caused by the deep network structure.
(2) The proposed MAFF module exploits the attention mechanism to selectively extract feature information of each scale, which can gain a global contextual view. The fusion of useful multi-scale features further improves the accuracy of brain tumor segmentation.
(3) MAFF-ResUNet performs well on the public BraTs 2019 MRI dataset and has certain competitiveness in the field of brain tumor segmentation.

## Materials and Methods

### Dataset

**FIGURE 1:**
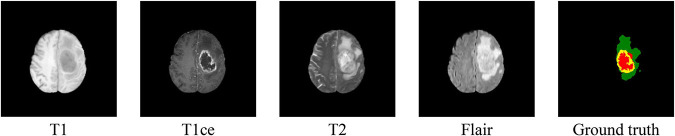
Samples of MRI images in four modalities and their ground truth. In the ground truth image, red, green, and yellow stand for tumor core (TC), whole tumor (WT), and enhance tumor (ET), respectively.

We performed our experiments on the MICCAI BraTs 2019 MRI dataset (Menze et al., 2015; Bakas et al., 2017a,b, 2018). The BraTs 2019 dataset is a collection of MRI data from glioma patients. There are two types of brain tumors in the dataset: high-grade glioma (HGG) and low-grade glioma (LGG). The dataset consists of 256 HGG cases and 76 LGG cases. Each case includes four 3D MRI modalities (T1, T1ce, T2, and Flair) as can be shown in Figure 1. And the size of each 3D MRI image is 155 × 240 × 240. The ground truth of each image is labeled manually by the expert. There are four types of labels: background (labeled 0), necrosis and non-enhancing tumor (labeled 1), edema (labeled 2), and enhancing tumor (labeled 4). The task is to segment three nested subregions generated by the three labels (1, 2, and 4), named enhancing tumor (ET, the region of label 4), whole tumor (WT, the region consists of label 1, 2, and 4) and tumor core (TC, the region of label 1 and 4).

### Preprocessing

In this work, 3D MRI images from 335 cases in the BraTs 2019 dataset are sliced into multiple 2D images, and slices without tumors are excluded. We use 80% of the generated slices for training, 10% for validation, and 10% for testing. Compared with single modal data, multi-modal data provide more characteristic information for tumor segmentation. To make effective use of multi-modal image information, we concatenate MRI 2D images of four modes in the same dimension as model input.

It is necessary to preprocess the input image before training. First, we remove the top 1% and bottom 1% intensities as Havaei did (Havaei et al., 2017). Then, since images of different modes have different contrast and other problems, we normalize each modal image before slicing. In this work, z-score normalization is adopted, that is, mean value and standard deviation are used to standardize each image. The formula is as follows:


(1) $$z_{i}=\frac{x_{i}-\mu }{\sigma }$$

where *x*_i_ is the input image, and *z*_i_ is the normalized image. μ represents the mean value of the input image, while σ denotes the standard deviation of the input image. Finally, we crop the training image into a size of 160 × 160 to reduce the black background in the image, which can obtain effective pixels and reduce the amount of calculation to some extent.

### Proposed Method

#### Architecture of MAFF-ResUNet

**FIGURE 2:**
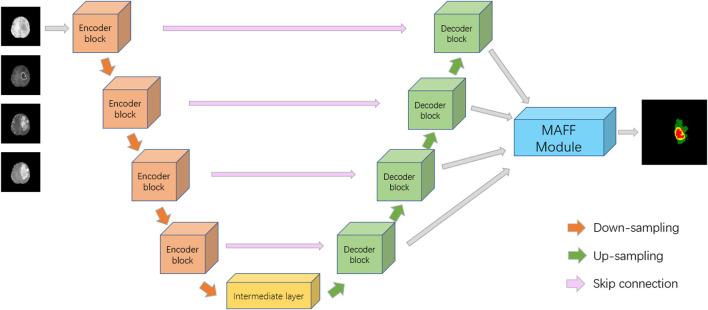
Architecture of the proposed MAFF-ResUNet. In the ground truth image, red, green and yellow stand for tumor core (TC), whole tumor (WT), and enhance tumor (ET), respectively.

Inspired by U-Net (Ronneberger et al., 2015), ResNet (He et al., 2016), DAF (Wang et al., 2018), we propose a deep convolutional neural network with a multi-scale attention feature fusion module based on attention mechanism for brain tumor segmentation. Recently, U-Net has achieved excellent performance in the field of medical image segmentation, which has the advantage of being able to accept input images of any size. Figure 2 shows the proposed MAFF-ResUNet, which adopts U-Net as our basic network architecture. The MAFF-ResUNet consists of four encoder blocks, four decoder blocks, an intermediate layer and a MAFF module. Firstly, in the down-sampling path, we utilize the convolution layer to extract low-level features of brain tumors, the pooling layer to expand the receptive field, and residual connections to enhance the expression ability of encoder blocks. In the up-sampling path, the up-sampling layer and convolution are used to restore the image resolution. Skip Connections combine low-level information with high-level information to reduce the loss of detailed information.

To further refine the boundaries of the brain tumor, we employ bilinear interpolation to up-sample the feature maps of different resolutions from the four decoder blocks to the same size as the input image and then input them into the MAFF module. The MAFF module extracts attention features of different scales and fuses them to improve the segmentation accuracy of brain tumors and obtain the segmentation results of brain tumors.

#### Encoder and Decoder Block

**FIGURE 3:**
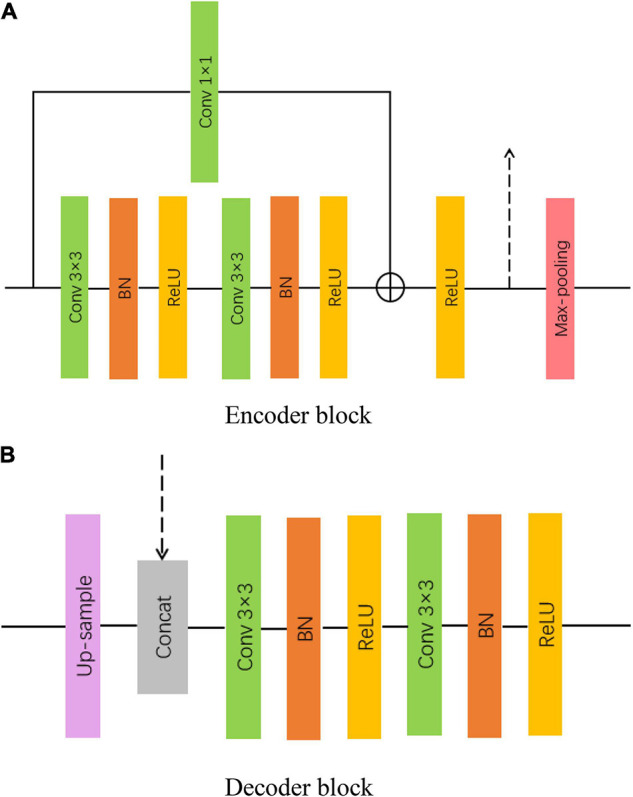
Encoder and decoder block in MAFF-ResUNet **(A)** encoder block **(B)** decoder block.

Residual U-Net is built by incorporating residual shortcuts into U-Net. Inspired by ResNet (He et al., 2016), we utilize five residual connections in the down-sampling branch, including four encoder blocks and an intermediate layer. The main function of these encoder blocks is to extract low-level features. The introduction of short skip connections is beneficial to obtain better feature expression and accelerate model convergence. As can be seen in Figure 3A, each encoder block contains two 3 × 3 convolutions, batch normalization (BN; Ioffe and Szegedy, 2015), Rectified Linear Unit (ReLU) activation function, and a 2 × 2 max-pooling layer. Each Max-pooling layer reduces the size of the input feature map to half of the original. Moreover, the intermediate layer plays the role of connecting the down-sampling and up-sampling paths. Structurally, the intermediate layer is similar to the encoder block, but without the pooling layer.

In the decoding stage, we use four decoder blocks, each of which contains an up-sampling layer, and two 3 × 3 convolutions (see Figure 3B). Similarly, each convolutional operation is followed by a BN layer and a ReLU activation layer. The up-sampling layer restores the size of the feature map by using the bilinear interpolation. The input of each decoder block is composed of two parts, one is the output of the previous decoder block, and the other is the output feature map of the same level encoder block, which makes up for the low-level details lost in the high-level semantic space.

#### Multi-Scale Attention Feature Fusion Module

**FIGURE 4:**
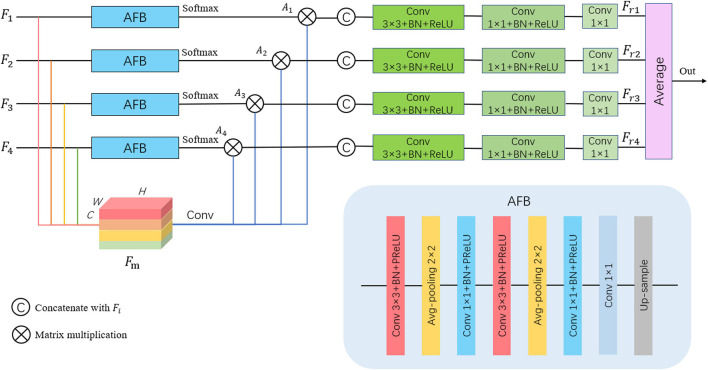
Multi-scale attention feature fusion (MAFF) module.

Inspired by DAF (Wang et al., 2018), we propose a MAFF module to fuse different scale features and improve the accuracy of brain tumor segmentation. As shown in Figure 4, the MAFF module accepts feature maps from four different scales, expressed as *F*_i_ ∈ *R*^*C*×*H*×*W*^(*i* = 1,2,3,4), where *i* indicates the feature map of the *i*-th level, *C* is the number of channels, *H* and *W* represent the height and width of *F*_i_, respectively. These four feature maps are concatenated in the channel dimension and named *F*_m_ ∈ *R*^4*C*×*H*×*W*^. The low-level feature map contains abundant boundary information of brain tumors, while the high-level feature map contains advanced semantic information of brain tumors.

The direct concatenation of different scale feature maps will certainly bring a little noise. The segmentation results obtained by directly exploiting *F*_m_ cannot effectively utilize the complementary information of features at different levels. Therefore, we use the attention feature block (AFB) to get the attention map *A*_i_ of each level and then multiply it with the mixed feature map *F*_m_ to obtain the redefined feature map *F*_ri_ ∈ *R*^*C*×*H*×*W*^ of each scale. Specifically, we do the following for *F*_i_:


(2) $$A_{i}=f_{s}\,\left(f_{u\,p}\,\left(g\,\left(f_{c\,a\,t}\,F_{i},f_{1\times 1}\,\left(F_{m}\right)\right)\right)\right)$$

where *f*_*1    ×1*_ is a 1 × 1 convolution followed by BN and ReLU function, *f*_cat_, *f*_up_, and *f*_s_ denote the operations of concatenation, up-sampling, and softmax function, respectively. The *g*(*x*) is an attention feature module composed of convolution and average pooling, which can be formulated as:


(3) $$g\,\left(x\right)=f_{1\times 1}\,\left(f_{p}\,\left(f_{3\times 3}\,\left(f_{p}\,\left(f_{3\times 3}\,\left(x\right)\right)\right)\right)\right)$$

where *f*_*3    ×3*_ is a convolution layer with the filter size of 3 × 3, and *f*_p_ represents the operation of average pooling. Besides, BN and parametric satisfaction linear Unit (PReLU; He et al., 2015) activation function are adopted after each convolution layer in the attention feature block. The PReLU can be obtained by:


(4) P⁢R⁢e⁢L⁢U⁢(x)=max(0,x)+a*min(0,x)

where *a* is a learnable parameter.

Then, the output of *g*(*x*) is fed to a softmax layer to obtain the attention map after the up-sample. The mathematical expression of the softmax function is given as:


(5) $$S\,o\,f\,t\,m\,a\,x\,\left(x_{j}\right)=\frac{e^{x_{j}}}{\sum_{n=1}^{N}e^{x_{n}}},j=1,2,\ldots ,n$$

According to the *A*_i_, we can selectively extract brain tumor-related feature information from the original feature map by performing a matrix multiplication between *A*_i_ and *F*_m_. The output maps are, respectively, concatenated with *F*_i_, and the convolution operation is performed to obtain the redefined feature maps.

In the end, the redefined feature maps of each layer containing both low-level and high-level information are fused, averaged, and then fed to a sigmoid function to obtain the final segmentation result.

#### Loss Function

In a specific task, the choice of a suitable loss function has a significant influence on the experimental results. The loss function is utilized to express the degree of difference between the predicted value and the label value. During the training process, the model continuously fine-tunes the weight and bias of the network to minimize the loss function value and improve the performance of the model. In this article, for the brain tumor segmentation task, our loss function consists of binary cross-entropy loss (BCE) and Dice loss. The Dice loss mainly applies the Dice coefficient, which is a similarity measurement function. The Dice loss takes the responsibility for the prediction of the brain tumor globally, while the BCE loss is responsible for the classification of each pixel. They can be expressed as:


(6) $$L\,o\,s\,s_{B\,C\,E}\,\left(p_{k},g_{k}\right)=-\frac{1}{n}\,\sum_{k}\left(g_{k}*\log\left(p_{k}\right)+\left(1-g_{k}\right)*\text{log}\,\left(1-p_{k}\right)\right)$$

(7) $$L\,o\,s\,s_{d\,i\,c\,e}\,\left(p_{k},g_{k}\right)=1-D\,i\,c\,e\,\left(p_{k},g_{k}\right)=1-\frac{2\,\left|p_{k}\,\cap g_{k}\right|+\varepsilon }{\left|p_{k}\right|+\left|g_{k}\right|+\varepsilon }$$

where *n* is the number of samples, *p*_k_ and *g*_k_ denote the prediction of the proposed model and the ground truth, respectively; |*p*_k_∩*g*_k_| represents the intersection between *p*_k_ and *g*_k_; |*p*_k_| and |*g*_k_| are the number of pixels in *p*_k_ and *g*_k_, respectively. ε stands for the smoothing coefficient, and the value is set to 1.0×10^−5^.

The total loss is described as:


(8) $$L\,o\,s\,s=\alpha \,L\,o\,s\,s_{B\,C\,E}+\beta \,L\,o\,s\,s_{d\,i\,c\,e}$$

where α and β represent the weight. We empirically set the weight α as 0.5, and the weight β as 1.

## Experiments and Results

### Training Details

The proposed MAFF-ResUNet is conducted on the PyTorch framework with an NVIDIA GeForce RTX 3090. In this experiment, we use adaptive moment estimation (Adam) (Kingma and Ba, 2014) as the optimizer. The initial learning rate is 0.0003, momentum is 0.90, and weight decay is set to 0.0001. We utilize poly police to decay the learning rate in the progress of training, as employed by Mou et al. (2019) and Elhassan et al. (2021). It can be defined as in Eq. (9), where *iter* represents the number of iterations, *max_iter* denotes the maximum number of iterations, and *power* is set to 0.9. During training, the batch size is 16.


(9) $$l\,r=l\,r_{i\,n\,i\,t}\times {\left(1-\frac{i\,t\,e\,r}{m\,a\,x\,\_\,i\,t\,e\,r}\right)}^{p\,o\,w\,e\,r}$$

### Evaluation Metrics

To effectively evaluate the performance of the proposed model, we adopt intersection-over-union (IoU), sensitivity, and positive predictive value (PPV), which are commonly used metrics for image segmentation. The IoU can be calculated using Eq. (10). (*P*∩*G*) is the number of positive pixels which values are the same in both *P* and *G*, while (*P*∪*G*) stands for the union of *P* and *G*. Sensitivity is defined as the ratio of correctly classified positive samples to the total positive samples in ground truth “*G*,” as shown in Eq. (11). It can be employed to measure the sensitivity of the model to segmentation targets. PPV represents the proportion of correctly classified positive samples to all positive samples in predicted “*P*,” which can be formulated as in Eq. (12). |*P*| is the number of positive pixels in *P*, while |*G*| is the number of positive pixels in *G*. The IoU, sensitivity, and PPV are all ranging from 0 to 1. The closer they are to 1, the better the segmentation result is:


(10) $$I\,o\,U\,\left(P,G\right)=\frac{\left(P\,\cap G\right)}{\left(P\,\cup G\right)}$$

(11) $$S\,E\,N\,\left(P,G\right)=\frac{\left(P\,\cap G\right)}{\left|G\right|}$$

(12) $$P\,P\,V\,\left(P,G\right)=\frac{\left(P\,\cap G\right)}{\left|P\right|}$$

Furthermore, as two commonly used metrics for brain tumor segmentation, the Dice similarity coefficient (DSC) and Hausdorff distance (HD) are also applied for the qualitative analysis in this experiment. The DSC is utilized to calculate how similar two samples are, and can be given as:


(13) $$D\,S\,C\,\left(P,G\right)=\frac{2\times \left|P\,\cap G\right|}{\left|P\right|+\left|G\right|}$$

The DSC is sensitive to the internal padding of the mask. Compared with the DSC, the HD is more sensitive to the segmented boundary. It represents the maximum Hausdorff distance between the labeled boundary and the predicted boundary, defined as:


$$H\,D\,\left(P,G\right)=\max\left\{d_{P\,G},d_{G\,P}\right\}$$

(14) $$=m\,a\,x\,\left\{\max_{p\in P}\min_{g\in G}\left(p,g\right),\max_{g\in G}\min_{p\in P}\left(g,p\right)\right\}$$

where *p* and g represent the points in the predicted area and the ground truth area, respectively.

### Performance Comparison

We compare the proposed MAFF-ResUNet with different networks, including FCN (Long et al., 2015) and U-Net (Ronneberger et al., 2015). For the FCN network, we will use three models with different network depths: FCN8s, FCN16s, FCN32s. As shown in Table 1, the various metrics of FCN in the brain tumor segmentation task are lower than that of other models. Compared with FCN, U-Net with encoder-decoder structure has stronger feature extraction capabilities, and the model performance is significantly improved. The MAFF-ResUNet proposed in this article, except for the PPV metric of TC slightly lower than U-Net, other metrics have improved.

**TABLE 1:** Comparison of segmentation results (mean ± SD) between the proposed MAFF-ResUNet and existing deep convolutional neural networks.

		**FCN32s**	**FCN16s**	**FCN8s**	**U-Net**	**MAFF-ResUNet**
IoU (%)	WT	72.90.3	77.80.4	80.80.2	85.60.4	**86.5 ± 0.08**
	TC	80.10.07	82.10.3	84.40.07	88.40.4	**88.5 ± 0.3**
	ET	66.70.6	73.10.2	77.70.05	85.80.4	**86.4 ± 0.3**

SEN (%)	WT	85.01.0	87.80.8	88.60.4	91.10.4	**91.93 ± 0.1**
	TC	87.60.4	89.70.3	90.50.05	92.60.3	**93.4 ± 0.1**
	ET	75.50.5	82.30.4	85.00.2	91.80.3	**92.5 ± 0.1**

PPV (%)	WT	81.70.6	85.30.3	88.30.08	92.00.09	**92.5 ± 0.1**
	TC	89.90.6	90.10.4	92.10.2	**94.4 ± 0.06**	93.9 ± 0.3
	ET	82.40.3	85.00.3	88.70.06	92.270.7	**92.34 ± 0.4**

DSC (%)	WT	81.90.3	85.20.3	87.30.2	90.50.3	**91.2 ± 0.06**
	TC	85.40.1	86.80.2	88.60.1	91.70.3	**91.8 ± 0.3**
	ET	73.00.6	79.40.2	83.40.09	89.80.4	**90.2 ± 0.3**

HD (mm)	WT	2.920.005	2.680.01	2.490.01	2.200.001	**2.16 ± 0.005**
	TC	1.730.005	1.640.006	1.560.003	1.410.01	**1.39 ± 0.006**
	ET	1.790.01	1.640.004	1.490.004	1.230.02	**1.20 ± 0.005**

*Bold indicates the maximum value of IoU, SEN, PPV, DSC, and the minimum value of HD among these methods.*

**FIGURE 5:**
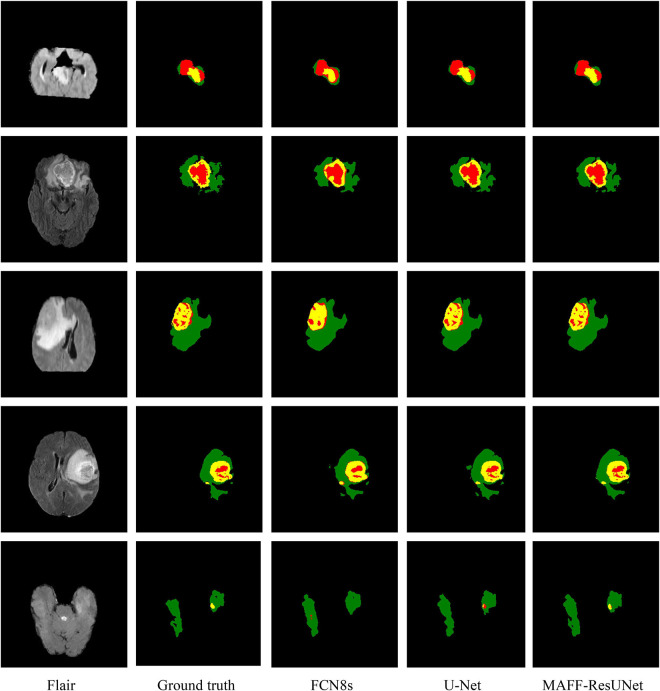
Visualized predicted images of different models. In the ground truth image, red, green and yellow represent tumor core (TC), whole tumor (WT), and enhance tumor (ET), respectively.

Moreover, we evaluate the performance of the proposed model from a more intuitive perspective. Figure 5 shows the comparison of the prediction image between the proposed approach in this article and other methods. The figure contains four different cases, and shows the original MRI images of flair modality, the prediction results of each model, and ground truth images. Since FCN8s has the best performance among the three FCN networks, we only use the prediction results of FCN8s for comparison. By comparison, we can find that the proposed method in this article is significantly better than U-Net and has obvious advantages in the segmentation of brain tumor contours and edge details. It shows that the introduction of the MAFF module makes the brain tumor segmentation results have richer edge information. Besides, there are fewer pixels mistakenly classified by the MAFF-ResUNet. The predicted images of the MAFF-ResUNet are more similar to manually annotated images.

## Conclusion

In this article, we propose a deep convolutional neural network for MRI brain tumor segmentation, named MAFF-ResUNet. This network takes advantage of the encoder-decoder structure. The introduction of residual shortcuts in the encoder block, combined with skip connections, enhances the global feature extraction capability of the network. In addition, for the output feature maps of different levels of decoder blocks, the attention mechanism is utilized to selectively extract important feature information of each level. Then the multi-scale feature maps are fused to obtain the segmentation. The proposed method is verified on the public BraTs 2019 MRI dataset. Experimental results show that the MAFF-ResUNet is better than existing deep convolutional neural networks. From the perspective of predicted images, the proposed method can effectively exploit multi-scale feature information and maintain most of the edge detail information. Therefore, the MAFF-ResUNet method proposed in this article can achieve high-precision automatic segmentation of brain tumors and can be used as an auxiliary tool for clinicians to perform early screening or diagnosis and treatment of brain tumors.

## Data Availability Statement

Publicly available datasets were analyzed in this study. This data can be found here: https://www.med.upenn.edu/cbica/brats-2019/.

## Ethics Statement

Ethical review and approval was not required for the study on human participants in accordance with the local legislation and institutional requirements. The patients/participants provided their written informed consent to participate in this study. Ethical review and approval was not required for the animal study because the data sets used in this manuscript are from public data sets. The data link is as follows: https://www.med.upenn.edu/cbica/brats-2019/. Written informed consent was obtained from the individual(s) for the publication of any potentially identifiable images or data included in this article.

## Author Contributions

XH wrote the main manuscript and conducted the experiments. WX and JY participated in the writing of the manuscript and modified the English grammar of the article. JY and SC made the experiments. JM and ZW analyzed the results. All authors reviewed the manuscript.

## Conflict of Interest

The authors declare that the research was conducted in the absence of any commercial or financial relationships that could be construed as a potential conflict of interest.

## Publisher’s Note

All claims expressed in this article are solely those of the authors and do not necessarily represent those of their affiliated organizations, or those of the publisher, the editors and the reviewers. Any product that may be evaluated in this article, or claim that may be made by its manufacturer, is not guaranteed or endorsed by the publisher.
